# Solitary wave behavior of (2+1)-dimensional Chaffee-Infante equation

**DOI:** 10.1371/journal.pone.0276961

**Published:** 2023-01-06

**Authors:** Saima Arshed, Ghazala Akram, Maasoomah Sadaf, Qurrat-ul-ain, Muhammad Bilal Riaz, Adam Wojciechowski

**Affiliations:** 1 Department of Mathematics, University of the Punjab, Quaid-e-Azam Campus, Lahore, Pakistan; 2 Faculty of Technical Physics, Information Technology and Applied Mathematics, Lodz University of Technology 90-924 Lodz Poland, Łódź, Poland; China University of Mining and Technology, CHINA

## Abstract

The behavior of gas diffusion in a homogeneous medium is described by the (2+1)-dimensional Chaffee-Infante equation. In this work, the solitary wave behavior of the (2+1)-dimensional Chaffee-Infante equation is studied with the help of extended sinh-Gordon equation expansion technique. Bright, dark, periodic, kink, anti-kink and singular traveling wave patterns are observed for suitable choice of parameters. The 3D graphs, 2D plots and contour plots are included to understand the dynamics of the obtained solutions. The obtained results depict that the extended sinh-Gordon equation expansion technique provides an efficient tool for solving other equations that occur in different branches of science and technology.

## 1 Introduction

Most of the nonlinear phenomena are described by partial differential equation in natural and applied sciences such as fluid dynamics, plasma physics, solid state physics, optical fibers, acoustics, biology and mathematical finance. The solutions of a wide range of nonlinear evolution equations exhibit the wave behavior corresponding to the underlying physical systems. In particular, solitary wave solutions and soliton solutions are of great interest for researchers owing to many applications in different areas of science. Significant work has been done in this area in recent years. Some of the recent explorations include studies on Schrödinger equation, Schrödinger-Maxwell-Bloch equation, coupled Hirota equations, Sawada-Kotera equation and others [1–4].

Many powerful techniques exist for obtaining solutions of partial differential equations in mathematical literature, such as; simple equations method [5], generalized projective Riccati equation method [6], first integral method [7], exp-function method [8], Bäcklund transformation method [9], sine-cosine method [10], homotopy analysis method [11], the (*G*′/*G*) expansion method [12] and $\bar{\partial }$-dressing method [13, 14].

One of the most important techniques to construct the exact traveling wave solutions is sinh-Gordon equation expansion technique. The present paper examines the (2+1)-dimensional Chaffee-Infante (CI) equation through extended sinh-Gordon equation expansion technique (shGEET) [15–17] for finding solitary wave solutions.

The CI equation is considered, as [18]

$$\begin{array}{c} v_{xt}+{\left(-v_{xx}+\gamma v^{3}-\gamma v\right)}_{x}+\delta v_{yy}=0, \end{array}$$ (1)

where *γ* is the coefficient of diffusion and *δ* is degradation coefficient.

The CI equation is a well-known reaction duffing equation [19, 20]. In any homogeneous medium the diffusion of a gas plays an important role in physical context and a useful model to study such phenomena is CI equation.

The rest of this article is organized as follows: Section 1 presents the description of method. Section 2 describes the formulation of solutions via sinh-Gordon equation expansion method. Section 3 explains the graphical representations. In Section 4, the conclusion is drawn.

## 2 Description of the extended shGEET

The general term of nonlinear partial differential equation is considered, as

$$\begin{array}{c} E(v,v_{x},v_{y},v_{t},v_{xx},v_{xy},v_{xt},v_{yt},v_{xxx},...)=0, \end{array}$$ (2)

where *v* = *v*(*x*, *y*, *t*).

The wave transformation is considered, as

$$\begin{array}{c} v=h(\zeta ),\;\;\;\;\;\;\zeta =\beta x+\epsilon y-\nu t. \end{array}$$ (3)

Transformation of Eq (3) is implemented on Eq (2), to obtain nonlinear ordinary differential equation ODE, as

$$\begin{array}{c} F(h,h^{\prime },h^{\prime \prime },...)=0, \end{array}$$ (4)

where ordinary derivatives with respect to *ζ* are indicated by “′”. *F* is a polynomial in *h* and its derivatives. Eq (4) is integrated, if possible, one or more times term by term. Consider the formal solutions of Eq (4) as follows:

$$\begin{array}{c} h\left(\omega \right)=\sum_{i=1}^{n}{\text{cosh}}^{i-1}\left(\omega \right){\left[b_{i}\text{sinh}\left(\omega \right)+a_{i}\text{cosh}\left(\omega \right)\right]}^{i}+a_{0}, \end{array}$$ (5)

where *ω*(*ζ*) satisfies the following equation [21].

$$\begin{array}{c} \omega^{\prime }=\sqrt{c+d{\text{sinh}}^{2}\left(\omega \right)}. \end{array}$$ (6)

The following cases arise after substituting different values of parameters *c* and *d* in Eq (6).

**Case 1**: Simplified form of sinh-Gordon equation is obtained after taking *c* = 0 and *d* = 1 in Eq (6)

$$\begin{array}{c} \omega^{\prime }=\text{sinh}\left(\omega \right). \end{array}$$ (7)

Simplifying Eq (7) [22], the solutions are obtained, as

$$\begin{array}{c} \text{sinh}(\omega )=\pm \iota \text{sech}(\zeta ),\;\;\;\;\text{cosh}(\omega )=-\text{tanh}(\zeta ) \end{array}$$ (8)

and

$$\begin{array}{c} \text{sinh}(\omega )=\pm \text{csch}(\zeta ),\;\;\;\;\text{cosh}(\omega )=-\text{coth}(\zeta ), \end{array}$$ (9)

where $\iota =\sqrt{-1}$.

The solution of Eq (5) along with Eqs (7), (8) and (9) is considered, as

$$\begin{array}{c} h\left(\zeta \right)=\sum_{i=1}^{n}{\left(-\text{tanh}\left(\zeta \right)\right)}^{i-1}{\left[\pm \iota b_{i}\text{sec}h\left(\zeta \right)-a_{i}\text{tanh}\left(\zeta \right)\right]}^{i}+a_{0} \end{array}$$ (10)

and

$$\begin{array}{c} h\left(\zeta \right)=\sum_{i=1}^{n}{\left(-\text{coth}\left(\zeta \right)\right)}^{i-1}{\left[\pm b_{i}\text{csc}h\left(\zeta \right)-a_{i}\text{coth}\left(\zeta \right)\right]}^{i}+a_{0}. \end{array}$$ (11)

**Case 2**: After taking *c* = 1 and *d* = 1, Eq (6) becomes

$$\begin{array}{c} \omega^{\prime }=\text{cosh}\left(\omega \right). \end{array}$$ (12)

This is also a simplified form of the sinh-Gordon equation. Simplifying Eq (12), the following solutions are obtained, as

$$\begin{array}{c} \text{sinh}(\omega )=\text{tan}(\zeta ),\;\;\;\;\text{cosh}(\omega )=\pm \text{sec}(\zeta ), \end{array}$$ (13)

$$\begin{array}{c} \text{sinh}(\omega )=-\text{cot}(\zeta ),\;\;\;\;\text{cosh}(\omega )=\pm \text{csc}(\zeta ). \end{array}$$ (14)

The solution of Eq (5) along with Eqs (12), (13) and (14) is considered, as

$$\begin{array}{c} h\left(\zeta \right)=\sum_{i=1}^{n}{\left(\pm \text{sec}\left(\zeta \right)\right)}^{i-1}{\left[b_{i}\text{tan}\left(\zeta \right)\pm a_{i}\text{sec}\left(\zeta \right)\right]}^{i}+a_{0}, \end{array}$$ (15)

$$\begin{array}{c} h\left(\zeta \right)=\sum_{i=1}^{n}{\left(\pm \text{csc}\left(\zeta \right)\right)}^{i-1}{\left[-b_{i}\text{cot}\left(\zeta \right)\pm a_{i}\text{csc}\left(\zeta \right)\right]}^{i}+a_{0}. \end{array}$$ (16)

The balancing number *n* is calculated by making balance between the highest order nonlinear term and highest order derivative term. A nonlinear algebraic system is determined by substituting the value of *n* in Eq (5) and using it along with Eq (6). Then setting the coefficients of sinh(*w*)^*j*^cosh(*w*)^*i*^, (*i* = 0, 1, 2, …, *j* = 0, 1), equal to zero and solving the given system the values of *a*_*i*_, *b*_*i*_, *ν*, *ϵ* and *β* are obtained. Finally plugging these values into Eqs (10) and (11) the required solutions are obtained. Similarly, Case 2 can be proceeded.

## 3 Formulation of the solutions

In this section, mathematical analysis of CI equation is given and its solutions are constructed along with cases arising in Section 1.

### 3.1 The mathematical analysis of CI equation

The traveling wave transformation is taken, as

$$\begin{array}{c} v(x,y,t)=h(\zeta ),\;\;\;\;\;\;\zeta =\beta x+\epsilon y-\nu t, \end{array}$$ (17)

where *ν* is wave velocity and *β* and *ϵ* are arbitrary unknowns. By applying the transformation Eq (17) into Eq (1), an ODE is obtained, as

$$\begin{array}{c} -\beta \gamma h^{\prime }+3\beta \gamma h^{2}h^{\prime }-\nu \beta h^{\prime \prime }+\epsilon^{2}\delta h^{\prime \prime }-\beta^{3}h^{\prime \prime \prime }=0. \end{array}$$ (18)

Integrating Eq (18) with respect to *ζ* and neglecting the constant of integration, yields

$$\begin{array}{c} -\beta \gamma h+\beta \gamma h^{3}-\nu \beta h^{\prime }+\epsilon^{2}\delta h^{\prime }-\beta^{3}h^{\prime \prime }=0. \end{array}$$ (19)

Balancing the power of *h*^3^ and $h^{{}^{\prime \prime }}$ produces the algebraic equation 3*n* = *n*+ 2 and simplification of this equation *n* = 1 is obtained.

#### 3.1.1 Case 1

In this case *ω*′ = sinh(*ω*). According to the extended shGEET [21], Eq (19) has the solutions of the form

$$\begin{array}{c} h\left(\zeta \right)=\left[\pm \iota b_{1}\text{sec}h\left(\zeta \right)-a_{1}\text{tanh}\left(\zeta \right)\right]+a_{0} \end{array}$$ (20)

and

$$\begin{array}{c} h\left(\zeta \right)=\left[\pm b_{1}\text{csc}h\left(\zeta \right)-a_{1}\text{coth}\left(\zeta \right)\right]+a_{0}. \end{array}$$ (21)

Hence, Eq (5) gives

$$\begin{array}{c} h\left(\omega \right)=b_{1}\text{sinh}\left(\omega \right)+a_{1}\text{cosh}\left(\omega \right)+a_{0}, \end{array}$$ (22)

where either *a*_1_ or *b*_1_ may be zero, but both *a*_1_ and *b*_1_ cannot be zero simultaneously. The nonlinear algebraic system is obtained by putting Eq (22) along with Eq (6) into Eq (19). The solution of the system provides the following values of the unknowns.

**Set 1**: *a*_0_ = 0, $a_{1}=-\iota \sqrt{2}$, *b*_1_ = 0, $\beta =-\iota \sqrt{\gamma }$, $\nu =\frac{\iota \epsilon^{2}\delta }{\gamma }$.

Putting the values from Set 1 into Eqs (20) and (21), the hyperbolic solutions for CI equation are obtained, as

$$\begin{array}{c} v_{11}=\sqrt{2}\text{sech}(-\frac{i\delta t\epsilon^{2}}{\sqrt{\gamma }}-i\sqrt{\gamma }x+\epsilon \text{y}) \end{array}$$ (23)

and

$$\begin{array}{c} v_{21}=-i\sqrt{2}\text{csch}(-\frac{i\delta t\epsilon^{2}}{\sqrt{\gamma }}-i\sqrt{\gamma }x+y\epsilon ). \end{array}$$ (24)

**Set 2**: $a_{0}=\frac{-1}{2}$, $a_{1}=\frac{-1}{2}$, $b_{1}=\frac{-1}{2}$, $\nu =\frac{3\gamma^{3/2}-2\sqrt{2}\epsilon^{2}\delta }{2\sqrt{\gamma }}$, $\beta =-\sqrt{\frac{\gamma }{2}}$.

Putting the values from Set 2 into Eqs (20) and (21), the solutions for CI equation are obtained, as

$$\begin{array}{ccc} v_{12} & = & \frac{1}{2}\text{tanh}(-\frac{t(3\gamma^{3/2}-2\sqrt{2}\epsilon^{2}\delta )}{2\sqrt{\gamma }}+\epsilon y-\sqrt{\frac{\gamma }{2}}x)\\ & & -\frac{1}{2}i\text{sech}(-\frac{t(3\gamma^{3/2}-2\sqrt{2}\epsilon^{2}\delta )}{2\sqrt{\gamma }}+\epsilon y-\sqrt{\frac{\gamma }{2}}x)-\frac{1}{2}\;\;\;\;\; \end{array}$$ (25)

and

$$\begin{array}{ccc} v_{22} & = & \frac{1}{2}\text{coth}(\epsilon y-\frac{t(3\gamma^{3/2}-2\sqrt{2}\delta \epsilon^{2})}{2\sqrt{\gamma }}-\sqrt{\frac{\gamma }{2}}x)\\ & & -\frac{1}{2}\text{csch}(\epsilon y-\frac{t(3\gamma^{3/2}-2\sqrt{2}\delta \epsilon^{2})}{2\sqrt{\gamma }}-\sqrt{\frac{\gamma }{2}}x)-\frac{1}{2}.\;\;\;\;\; \end{array}$$ (26)

**Set 3**: *a*_0_ = 0, *a*_1_ = −1, *b*_1_ = −1, $\nu =\frac{\epsilon^{2}\delta }{\sqrt{2\gamma }}$, $\beta =\sqrt{2\gamma }$.

Putting the values from Set 3 into Eqs (20) and (21), the solutions for CI equation are obtained, as

$$\begin{array}{c} v_{13}=\text{tanh}(-\frac{\delta t\epsilon^{2}}{\sqrt{2\gamma }}+\sqrt{2\gamma }x+y\epsilon )-i\text{sech}(-\frac{\delta t\epsilon^{2}}{\sqrt{2\gamma }}+\sqrt{2\gamma }x+y\epsilon ) \end{array}$$ (27)

and

$$\begin{array}{c} v_{23}=\text{coth}(-\frac{\delta t\epsilon^{2}}{\sqrt{2\gamma }}+\sqrt{2\gamma }x+y\epsilon )-\text{csch}(-\frac{\delta t\epsilon^{2}}{\sqrt{2\gamma }}+\sqrt{2\gamma }x+y\epsilon ). \end{array}$$ (28)

**Set 4**: $a_{0}=\frac{1}{2}$, $a_{1}=\frac{-1}{2}$, $b_{1}=\frac{1}{2}$, $\nu =(\frac{3\gamma^{3/2}+2\sqrt{2}\epsilon^{2}\delta }{2\sqrt{\gamma }})$, $\beta =-\sqrt{\frac{\gamma }{2}}$.

Putting the values from Set 4 into Eqs (20) and (21), the solutions for CI equation are obtained, as

$$\begin{array}{ccc} v_{14} & = & -\frac{1}{2}\text{tanh}(-\frac{t(3\gamma^{3/2}+2\sqrt{2}\epsilon^{2}\delta )}{2\sqrt{\gamma }}+\epsilon y-\sqrt{\frac{\gamma }{2}}x)\\ & & -\frac{1}{2}i\text{sech}(-\frac{t(3\gamma^{3/2}+2\sqrt{2}\epsilon^{2}\delta )}{2\sqrt{\gamma }}-\sqrt{\frac{\gamma }{2}}x+y\epsilon )+\frac{1}{2} \end{array}$$ (29)

and

$$\begin{array}{ccc} v_{24} & = & -\frac{1}{2}\text{coth}(-\frac{t(3\gamma^{3/2}+2\sqrt{2}\epsilon^{2}\delta )}{2\sqrt{\gamma }}+\epsilon y-\sqrt{\frac{\gamma }{2}}x)\\ & & -\frac{1}{2}\text{csch}(-\frac{t(3\gamma^{3/2}+2\sqrt{2}\epsilon^{2}\delta )}{2\sqrt{\gamma }}+\epsilon y-\sqrt{\frac{\gamma }{2}}x)+\frac{1}{2}. \end{array}$$ (30)

**Set 5**: *a*_0_ = 0, *a*_1_ = 0, *b*_1_ = 1, $\nu =-\sqrt{\frac{2}{\gamma }}\epsilon^{2}\delta$, $\beta =-\sqrt{\frac{\gamma }{2}}$.

Putting the values from Set 5 into Eqs (20) and (21), the solutions for CI equation are obtained, as

$$\begin{array}{c} v_{15}=-\text{tanh}(\frac{\delta t\epsilon^{2}}{\sqrt{\gamma }}-\frac{\sqrt{\gamma }x}{\sqrt{2}}+y\epsilon ) \end{array}$$ (31)

and

$$\begin{array}{c} v_{25}=-\text{coth}(\frac{\sqrt{2}\delta t\epsilon^{2}}{\sqrt{\gamma }}-\frac{\sqrt{\gamma }x}{\sqrt{2}}+y\epsilon ). \end{array}$$ (32)

**Set 6**: $a_{0}=\frac{-1}{2}$, *a*_1_ = 0, $b_{1}=\frac{-1}{2}$, $\nu =\frac{3\gamma^{3/2}-8\sqrt{2}\epsilon^{2}\delta }{4\sqrt{\gamma }}$, $\beta =-\frac{\sqrt{\gamma }}{2^{3/2}}$.

Putting the values from Set 6 into Eqs (20) and (21), the solutions for CI equation are obtained, as

$$\begin{array}{c} v_{16}=\frac{1}{2}\text{tanh}(-\frac{t(3\gamma^{3/2}-8\sqrt{2}\epsilon^{2}\delta )}{4\sqrt{\gamma }}+\epsilon y-\frac{\sqrt{\gamma }x}{2^{3/2}})-\frac{1}{2} \end{array}$$ (33)

and

$$\begin{array}{c} v_{26}=\frac{1}{2}\text{coth}(-\frac{t(3\gamma^{3/2}-8\sqrt{2}\epsilon^{2}\delta )}{4\sqrt{\gamma }}+\epsilon y-\frac{\sqrt{\gamma }x}{2^{3/2}})-\frac{1}{2}. \end{array}$$ (34)

**Set 7**: $a_{0}=\frac{-1}{2}$, *a*_1_ = 0, $b_{1}=\frac{1}{2}$, $\nu =\frac{-3\gamma^{3/2}-8\sqrt{2}\epsilon^{2}\delta }{4\sqrt{\gamma }}.$, $\beta =\frac{\sqrt{\gamma }}{2^{3/2}}$.

Putting the values from Set 7 into Eqs (20) and (21), the solutions for CI equation are obtained, as

$$\begin{array}{c} v_{17}=-\frac{1}{2}\text{tanh}(-\frac{t(-3\gamma^{3/2}-8\sqrt{2}\epsilon^{2}\delta )}{4\sqrt{\gamma }}+\epsilon y+\frac{\sqrt{\gamma }x}{2\sqrt{2}})-\frac{1}{2} \end{array}$$ (35)

and

$$\begin{array}{c} v_{27}=-\frac{1}{2}\text{coth}(-\frac{t(-3\gamma^{3/2}-8\sqrt{2}\epsilon^{2}\delta )}{4\sqrt{\gamma }}+\epsilon y+\frac{\sqrt{\gamma }x}{2\sqrt{2}})-\frac{1}{2}. \end{array}$$ (36)

#### 3.1.2 Case 2

In this case *ω*′ = cosh(*ω*). According to the extended shGEET [21], Eq (19) has solutions of the form

$$\begin{array}{c} h\left(\zeta \right)=\left[b_{1}\text{tan}\left(\zeta \right)\pm a_{1}\text{sec}\left(\zeta \right)\right]+a_{0} \end{array}$$ (37)

and

$$\begin{array}{c} h\left(\zeta \right)=\left[-b_{1}\text{cot}\left(\zeta \right)\pm a_{1}\text{csc}\left(\zeta \right)\right]+a_{0} \end{array}$$ (38)

and therefore Eq (5) gives

$$\begin{array}{c} h\left(\omega \right)=b_{1}\text{sinh}\left(\omega \right)+a_{1}\text{cosh}\left(\omega \right)+a_{0}, \end{array}$$ (39)

where either *a*_1_ or *b*_1_ may be zero, but both *a*_1_ and *b*_1_ cannot be zero simultaneously. The nonlinear algebraic system is obtained after putting Eq (39) along with Eq (6) into Eq (19). The solution of the system provides the following values of the unknowns.

**Set 1**: *a*_0_ = 0, *a*_1_ = 0, $b_{1}=\sqrt{2}$, $\beta =-\sqrt{\gamma }$, $\nu =-\frac{\epsilon^{2}\delta }{\sqrt{\gamma }}$.

Putting the values from Set 1 into Eqs (37) and (38), the solutions for CI equation are obtained, as

$$\begin{array}{c} v_{31}=\sqrt{2}\text{sec}(\frac{\epsilon^{2}\delta t}{\sqrt{\gamma }}+\epsilon y-\sqrt{\gamma }x) \end{array}$$ (40)

and

$$\begin{array}{c} v_{41}=\sqrt{2}\text{csc}(\frac{\epsilon^{2}\delta t}{\sqrt{\gamma }}+\epsilon y-\sqrt{\gamma }x). \end{array}$$ (41)

**Set 2**: $a_{0}=\frac{-1}{2}$, $a_{1}=\frac{-1}{2}$, $b_{1}=\frac{-1}{2}$, $\nu =\frac{i(3\gamma^{3/2}-8\sqrt{2}\epsilon^{2}\delta )}{4\sqrt{\gamma }}$, $\beta =\frac{i\sqrt{\gamma }}{2\sqrt{2}}$.

Putting the values from Set 2 into Eqs (37) and (38), the solutions for CI equation are obtained, as

$$\begin{array}{c} v_{32}=-\frac{1}{2}-\frac{1}{2}i\text{tan}(-\frac{it(3\gamma^{3/2}-8\sqrt{2}\epsilon^{2}\delta )}{4\sqrt{\gamma }}+\epsilon y+\frac{i\sqrt{\gamma }x}{2\sqrt{2}}) \end{array}$$ (42)

and

$$\begin{array}{c} v_{42}=-\frac{1}{2}+\frac{1}{2}i\text{cot}(-\frac{it(3\gamma^{3/2}-8\sqrt{2}\epsilon^{2}\delta )}{4\sqrt{\gamma }}+\epsilon y+\frac{i\sqrt{\gamma }x}{2\sqrt{2}}). \end{array}$$ (43)

**Set 3**: *a*_0_ = 0, *a*_1_ = −1, *b*_1_ = −1, $\nu =\frac{i(3\gamma^{3/2}+2\sqrt{2}\epsilon^{2}\delta )}{2\sqrt{\gamma }}$, $\beta =-\frac{i\sqrt{\gamma }}{\sqrt{2}}$.

Putting the values from Set 3 into Eqs (37) and (38), the solitary wave solutions for CI equation are obtained, as

$$\begin{array}{ccc} v_{33} & = & -\frac{1}{2}i\text{tan}(-\frac{it(3\gamma^{3/2}+2\sqrt{2}\epsilon^{2}\delta )}{2\sqrt{\gamma }}+\epsilon y-\frac{i\sqrt{\gamma }x}{\sqrt{2}})\\ & & -\frac{1}{2}i\text{sec}(-\frac{it(3\gamma^{3/2}+2\sqrt{2}\epsilon^{2}\delta )}{2\sqrt{\gamma }}+\epsilon y-\frac{i\sqrt{\gamma }x}{\sqrt{2}})-\frac{1}{2} \end{array}$$ (44)

and

$$\begin{array}{ccc} v_{43} & = & \frac{1}{2}i\text{cot}(-\frac{it(3\gamma^{3/2}+2\sqrt{2}\epsilon^{2}\delta )}{2\sqrt{\gamma }}+\epsilon y-\frac{i\sqrt{\gamma }x}{\sqrt{2}})\\ & & -\frac{1}{2}i\text{csc}(-\frac{it(3\gamma^{3/2}+2\sqrt{2}\epsilon^{2}\delta )}{2\sqrt{\gamma }}+\epsilon y-\frac{i\sqrt{\gamma }x}{\sqrt{2}})-\frac{1}{2}. \end{array}$$ (45)

**Set 4**: $a_{0}=\frac{1}{2}$, $a_{1}=\frac{-\iota }{2}$, $b_{1}=\frac{-\iota }{2}$, $\nu =-\frac{i(3\gamma^{3/2}-2\sqrt{2}\epsilon^{2}\delta )}{2\sqrt{\gamma }}$, $\beta =-\iota \sqrt{\frac{\gamma }{2}}$.

Putting the values from Set 4 into Eqs (37) and (38), the solitary wave solutions for CI equation are obtained, as

$$\begin{array}{ccc} v_{34} & = & -\frac{1}{2}i\text{tan}(\frac{it(3\gamma^{3/2}-2\sqrt{2}\epsilon^{2}\delta )}{2\sqrt{\gamma }}+\epsilon y-\frac{i\sqrt{\gamma }x}{\sqrt{2}})\\ & & -\frac{1}{2}i\text{sec}(\frac{it(3\gamma^{3/2}-2\sqrt{2}\epsilon^{2}\delta )}{2\sqrt{\gamma }}+\epsilon y-\frac{i\sqrt{\gamma }x}{\sqrt{2}})+\frac{1}{2} \end{array}$$ (46)

and

$$\begin{array}{ccc} v_{44} & = & \frac{1}{2}i\text{cot}(\frac{it(3\gamma^{3/2}-2\sqrt{2}\epsilon^{2}\delta )}{2\sqrt{\gamma }}+\epsilon y-\frac{i\sqrt{\gamma }x}{\sqrt{2}})\\ & & +\frac{1}{2}i\text{csc}(\frac{it(3\gamma^{3/2}-2\sqrt{2}\epsilon^{2}\delta )}{2\sqrt{\gamma }}+\epsilon y-\frac{i\sqrt{\gamma }x}{\sqrt{2}})+\frac{1}{2}. \end{array}$$ (47)

**Set 5**: $a_{0}=\frac{-1}{2}$, $a_{1}=\frac{\iota }{2}$, *b*_1_ = 0, $\nu =-\frac{i(3\gamma^{3/2}+8\sqrt{2}\epsilon^{2}\delta )}{4\sqrt{\gamma }}$, $\beta =\frac{i\sqrt{\gamma }}{2\sqrt{2}}$.

Putting the values from Set 5 into Eqs (37) and (38), the solutions for CI equation are obtained, as

$$\begin{array}{c} v_{35}=-\frac{1}{2}+\frac{1}{2}i\text{tan}(\frac{it(3\gamma^{3/2}+8\sqrt{2}\epsilon^{2}\delta )}{4\sqrt{\gamma }}+\epsilon y+\frac{i\sqrt{\gamma }x}{2\sqrt{2}}) \end{array}$$ (48)

and

$$\begin{array}{c} v_{45}=-\frac{1}{2}-\frac{1}{2}i\text{cot}(\frac{it(3\gamma^{3/2}+8\sqrt{2}\epsilon^{2}\delta )}{4\sqrt{\gamma }}+\epsilon y+\frac{i\sqrt{\gamma }x}{2\sqrt{2}}). \end{array}$$ (49)

**Set 6**: *a*_0_ = 0, *a*_1_ = −*ι*, *b*_1_ = 0, $\nu =-\frac{i\sqrt{2}\epsilon^{2}\delta }{\sqrt{\gamma }}$, $\beta =\frac{i\sqrt{\gamma }}{\sqrt{2}}$.

Putting the values from Set 6 into Eqs (37) and (38), the solitary wave solutions for CI equation are obtained, as

$$\begin{array}{c} v_{36}=-i\text{tan}(\frac{\sqrt{2}i\epsilon^{2}\delta t}{\sqrt{\gamma }}+\epsilon y+\frac{i\sqrt{\gamma }x}{\sqrt{2}}) \end{array}$$ (50)

and

$$\begin{array}{c} v_{46}=i\text{cot}(\frac{\sqrt{2}i\epsilon^{2}\delta t}{\sqrt{\gamma }}+\epsilon y+\frac{i\sqrt{\gamma }x}{\sqrt{2}}). \end{array}$$ (51)

**Set 7**: *a*_0_ = 0, *a*_1_ = *ι*, *b*_1_ = −*ι*, $\nu =-\frac{i\epsilon^{2}\delta }{\sqrt{2\gamma }}$, $\beta =i\sqrt{2\gamma }$.

Putting the values from Set 7 into Eqs (37) and (38), the solutions for CI equation are obtained, as

$$\begin{array}{c} v_{37}=i\text{tan}(\frac{i\epsilon^{2}\delta t}{\sqrt{2\gamma }}+\epsilon y+i\sqrt{2\gamma }x)-i\text{sec}(\frac{i\epsilon^{2}\delta t}{\sqrt{2\gamma }}+\epsilon y+i\sqrt{2\gamma }x) \end{array}$$ (52)

and

$$\begin{array}{c} v_{47}=-i\text{cot}(\frac{i\epsilon^{2}\delta t}{\sqrt{2\gamma }}+\epsilon y+i\sqrt{2\gamma }x)-i\text{csc}(\frac{i\epsilon^{2}\delta t}{\sqrt{2\gamma }}+\epsilon y+i\sqrt{2\gamma }x). \end{array}$$ (53)

## 4 Graphical representation

In this section, the 3D surface graphs, 2D plots and the 2D contour plots are depicted to understand the dynamics of the obtained solutions.

**Fig 1:**
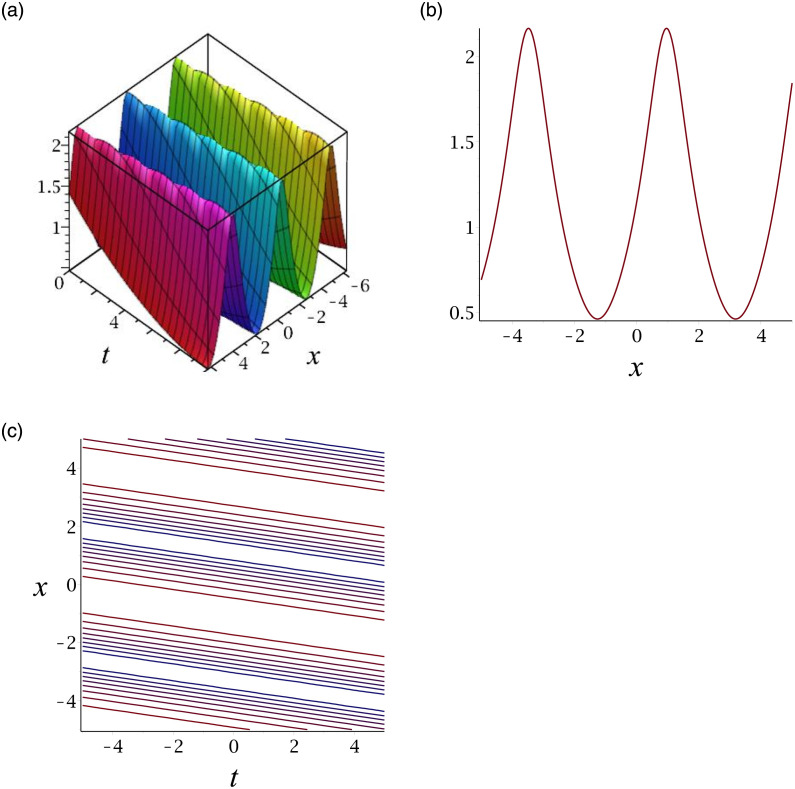
3D surface graphs, 2D plot and contour plot for |*v*_13_|.

Fig 1 depicts the solitary wave corresponding to |*v*_13_|. Subplot (a) demonstrates the surface graph by taking *γ* = −1, *ϵ* = 1, *δ* = 0.3, *y* = 1, (b) represents the propagation of the wave in the direction of *x* − *axis*, whereas (c) shows its contour.

**Fig 2:**
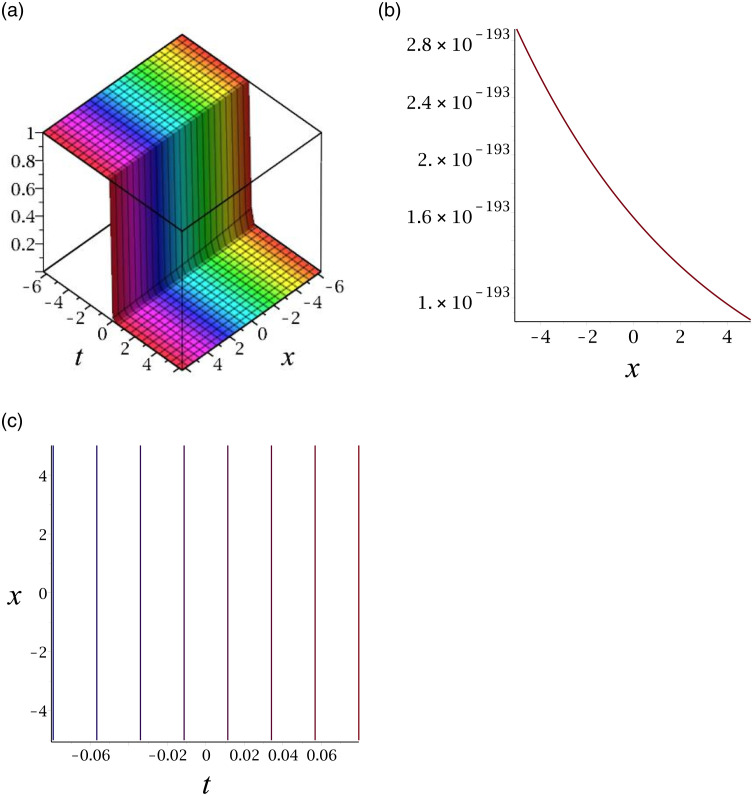
3D surface graphs, 2D plot and contour plot for |*v*_14_|.

Fig 2 depicts the kink shape soliton for |*v*_14_|. Subplot (a) demonstrates the surface by taking *γ* = 0.03, *ϵ* = 3, *δ* = 6, *y* = 1, (b) represents the propagation of the wave in the direction of *x* − *axis*, whereas (c) shows its contour.

**Fig 3:**
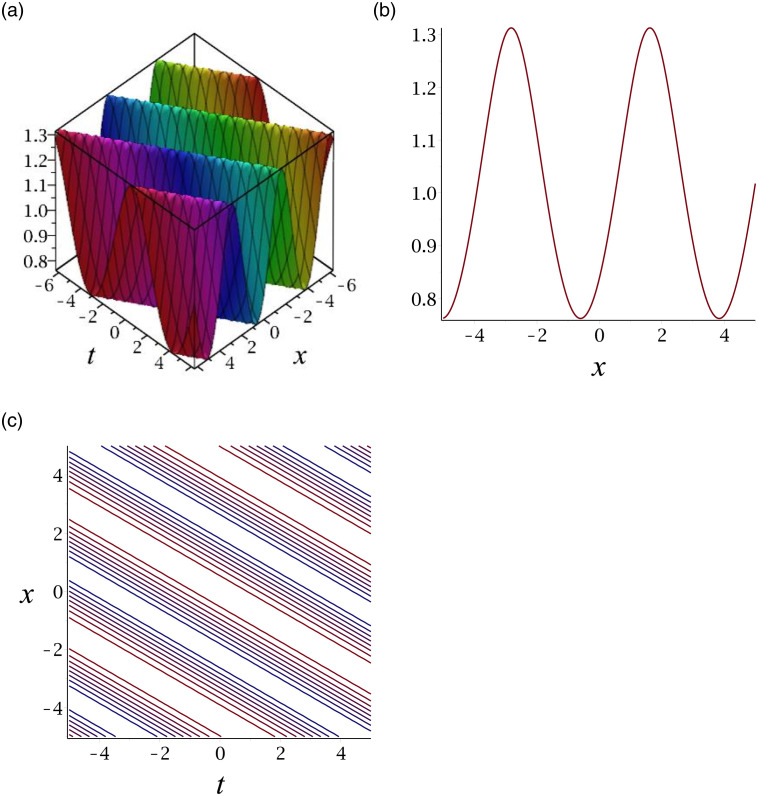
3D surface graphs, 2D plot and contour plot for |*v*_15_|.

Fig 3 depicts the periodic wave for |*v*_15_|. Subplot (a) demonstrates the surface by taking *γ* = −1, *ϵ* = 1, *δ* = 0.3, *y* = 1, (b) represents the propagation of the wave in the direction of *x* − *axis*, whereas (c) shows its contour.

**Fig 4:**
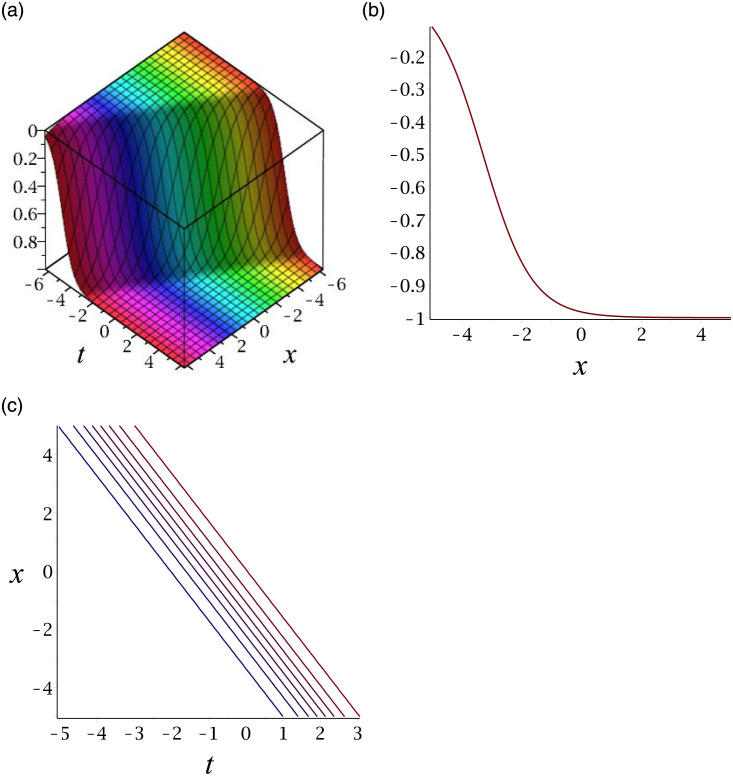
3D surface graphs, 2D plot and contour plot for *v*_17_.

Fig 4 depicts the kink soliton for the solution *v*_17_. Subplot (a) demonstrates the surface by taking *γ* = 3, *ϵ* = −2, *δ* = 1, *y* = 1, (b) represents the propagation of the wave in the direction of *x* − *axis*, whereas (c) shows its contour.

**Fig 5:**
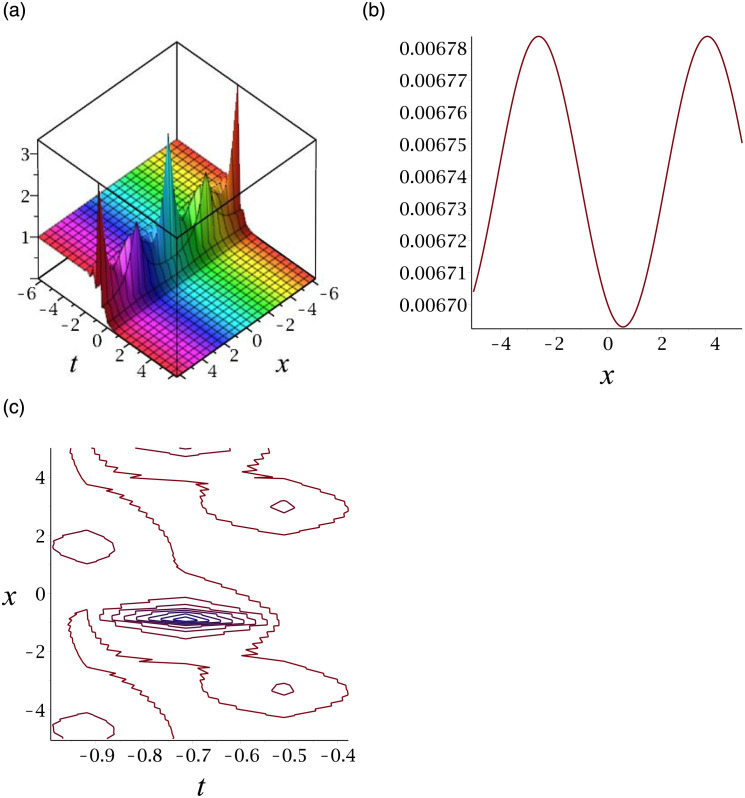
3D surface graphs, 2D plot and contour plot for |*v*_22_|.

Fig 5 depicts the solitary wave corresponding to |*v*_22_|. Subplot (a) demonstrates the surface by taking *γ* = −2, *ϵ* = 3, *δ* = 2, *y* = 1, (b) represents the propagation of the wave in the direction of *x* − *axis*, whereas (c) shows its contour.

**Fig 6:**
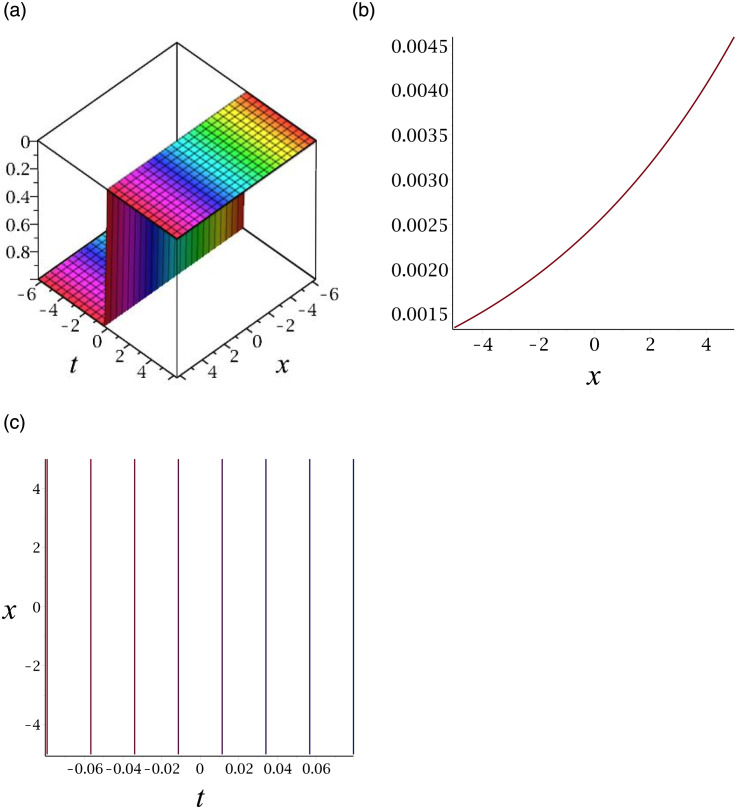
3D surface graphs, 2D plot and contour plot for *v*_26_.

Fig 6 depicts the anti-kink soliton for the solution *v*_26_. Subplot (a) demonstrates the surface by taking *γ* = 0.03, *ϵ* = 3, *δ* = 6, *y* = 1, (b) represents the propagation of the wave in the direction of *x* − *axis*, whereas (c) shows its contour.

**Fig 7:**
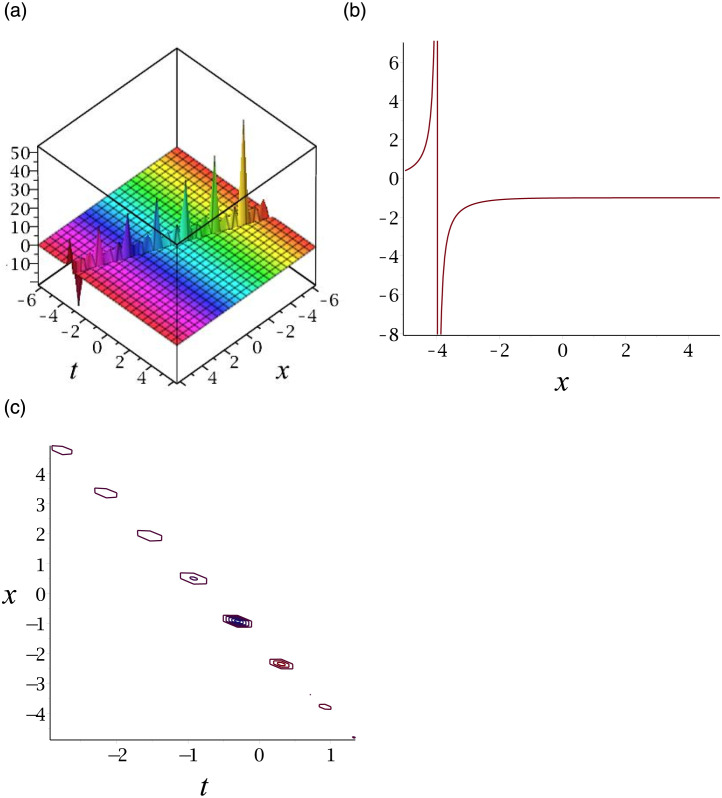
3D surface graphs, 2D plot and contour plot for *v*_27_.

The graphs of |*v*_16_|, |*v*_21_|, *v*_27_, |*v*_36_|, *v*_41_ and |*v*_46_| are singular solitons. For compactness, the graph of *v*_27_ is included. Fig 7 depicts the singular soliton for the solution *v*_27_. Subplot (a) demonstrates the surface by taking *γ* = 3, *ϵ* = 1, *δ* = −0.5, *y* = 1, (b) represents the propagation of the wave in the direction of *x* − *axis*, whereas (c) shows its contour.

**Fig 8:**
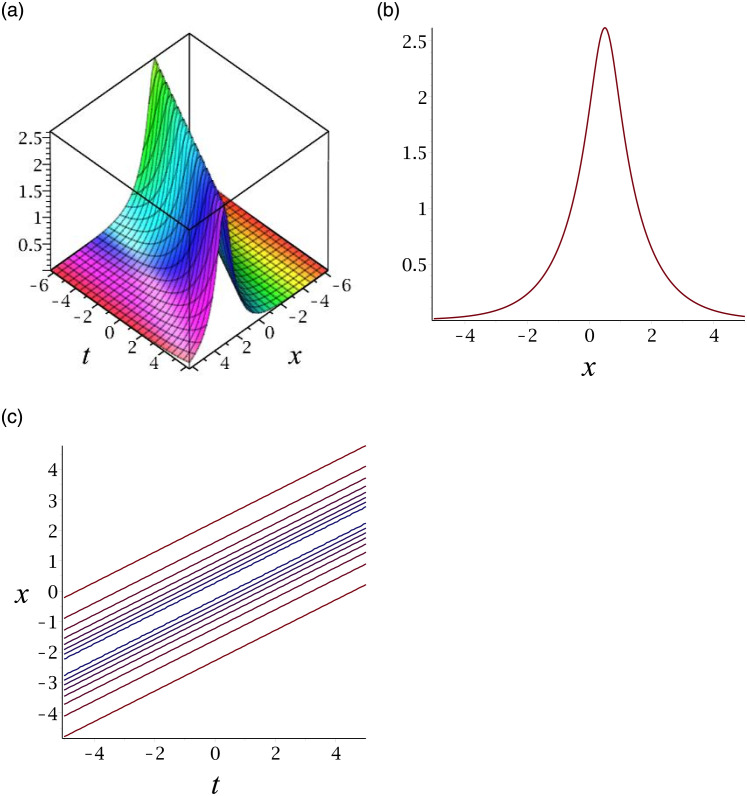
3D surface graphs, 2D plot and contour plot for |*v*_31_|.

The graphs of |*v*_11_| and |*v*_31_| show bright solitons. For compactness, the graph of |*v*_31_| is included. Fig 8 depicts the bright soliton for |*v*_31_|. Subplot (a) demonstrates the surface by taking *γ* = −1, *ϵ* = 1, *δ* = −0.5, *y* = 1, (b) represents the propagation of the wave in the direction of *x* − *axis*, whereas (c) shows its contour.

**Fig 9:**
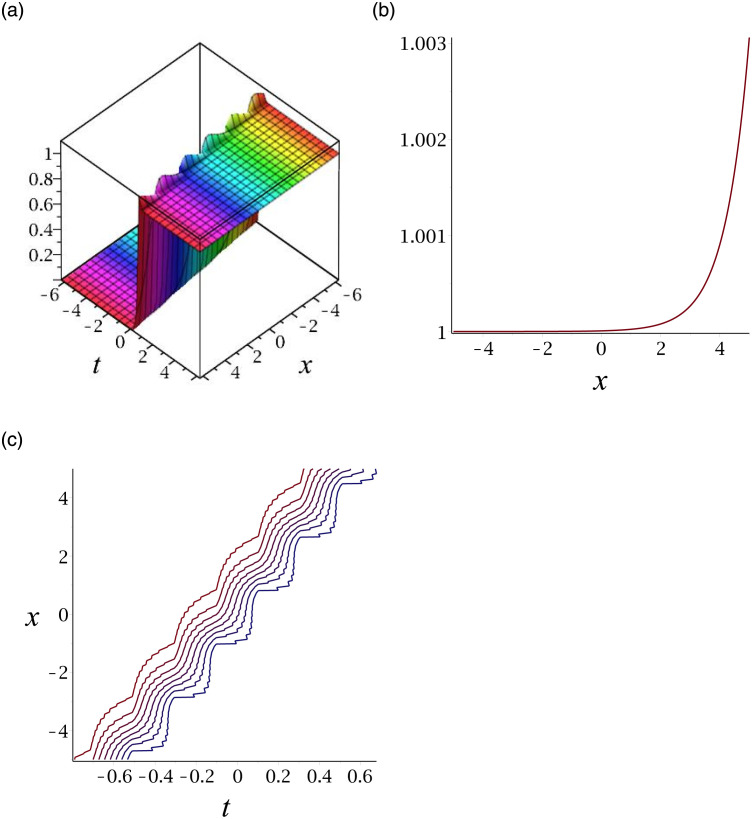
3D surface graphs, 2D plot and contour plot for |*v*_32_|.

Fig 9 depicts the graph of |*v*_32_|. Subplot (a) demonstrates the surface by taking *γ* = 3, *ϵ* = −2, *δ* = 1, *y* = 1, (b) represents the propagation of the wave in the direction of *x* − *axis*, whereas (c) shows its contour.

**Fig 10:**
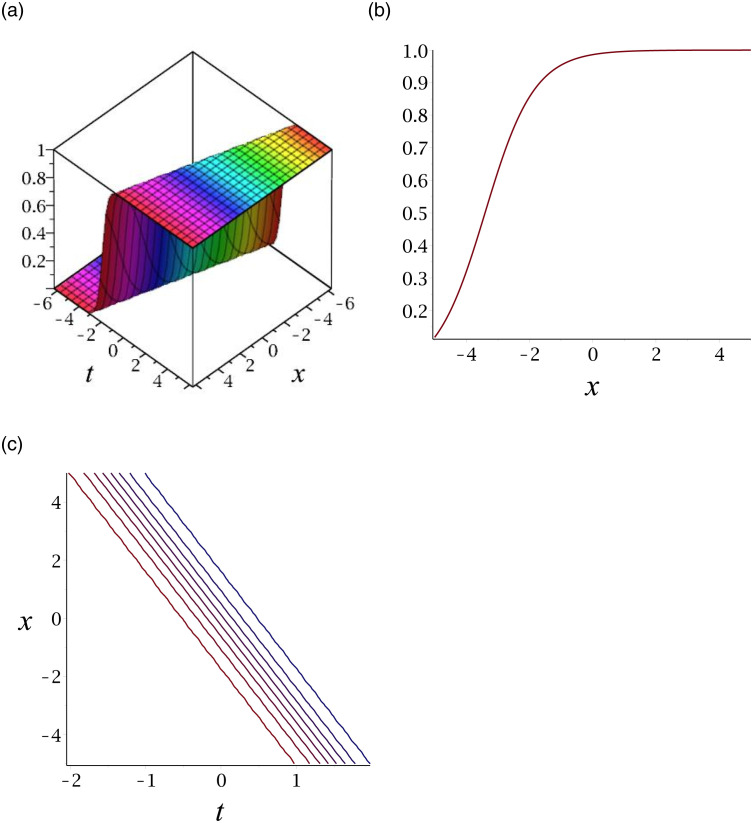
3D surface graphic and 2D plot and contour plot for |*v*_33_|.

Fig 10 depicts the anti-kink soliton solution of |*v*_33_|. Subplot (a) demonstrates the surface by taking *γ* = 3, *ϵ* = 1, *δ* = −0.5, *y* = 1, (b) represents the propagation of the wave in the direction of *x* − *axis*, whereas (c) shows its contour.

**Fig 11:**
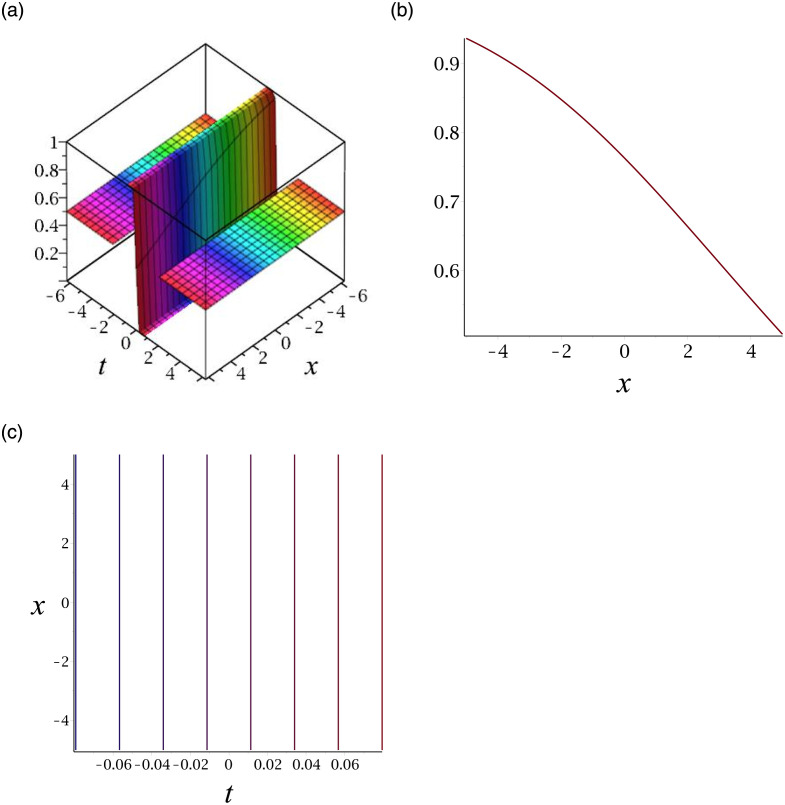
3D surface graphs, 2D plot and contour plot for |*v*_34_|.

The graphs of |*v*_34_| and |*v*_44_| are singular kink shape solitons. For compactness, the graph of |*v*_34_| is included. Fig 11 depicts the singular kink shape soliton solution of *v*_34_. Subplot (a) demonstrates the surface by taking *γ* = 0.03, *ϵ* = 3, *δ* = 5, *y* = 1, (b) represents the propagation of the wave in the direction of *x* − *axis*, whereas (c) shows its contour.

**Fig 12:**
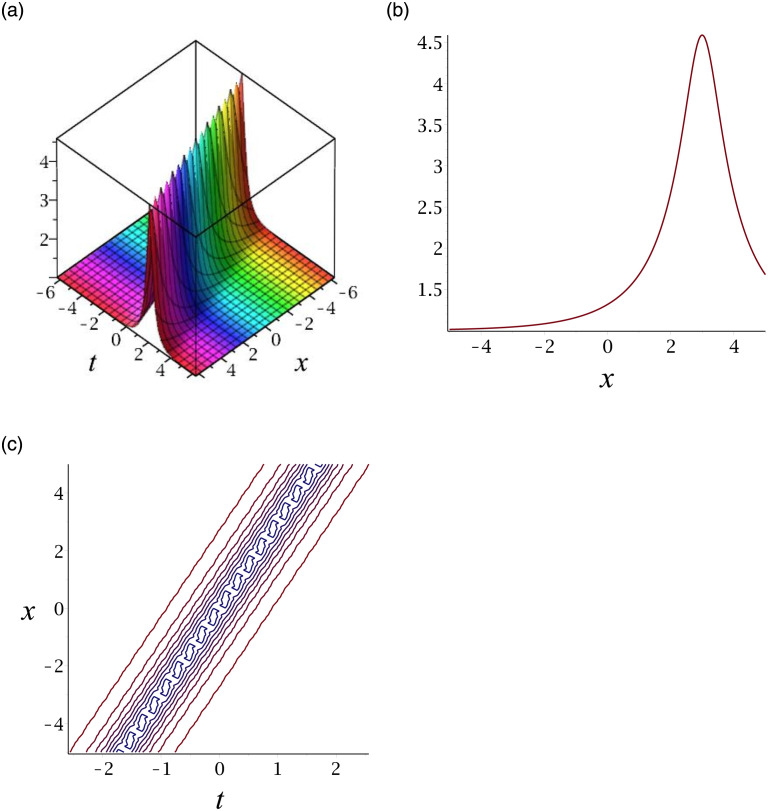
3D surface graphs, 2D plot and contour plot for |*v*_37_|.

Fig 12 depicts the bright shape soliton for |*v*_37_|. Subplot (a) demonstrates the surface by taking *γ* = 0.2, *ϵ* = 2, *δ* = −0.3, *y* = 1, (b) represents the propagation of the wave in the direction of *x* − *axis*, whereas (c) shows its contour.

**Fig 13:**
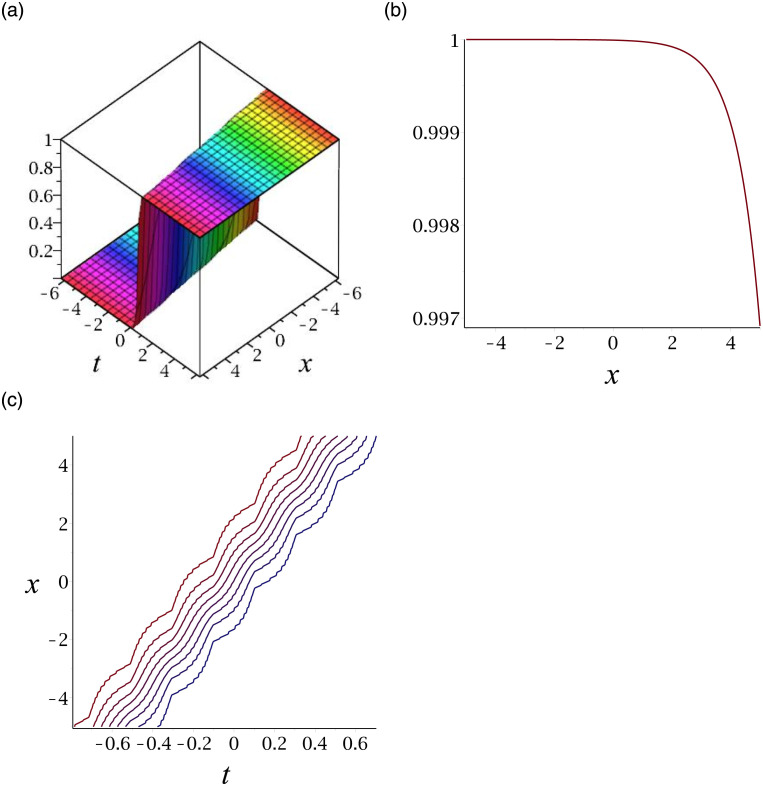
3D surface graphs, 2D plot and contour plot for |*v*_42_|.

Fig 13 depicts the anti-kink soliton corresponding to |*v*_42_|. Subplot (a) demonstrates the surface by taking *γ* = 3, *ϵ* = 1, *δ* = −2, *y* = 1, (b) represents the propagation of the wave in the direction of *x* − *axis*, whereas (c) shows its contour.

**Fig 14:**
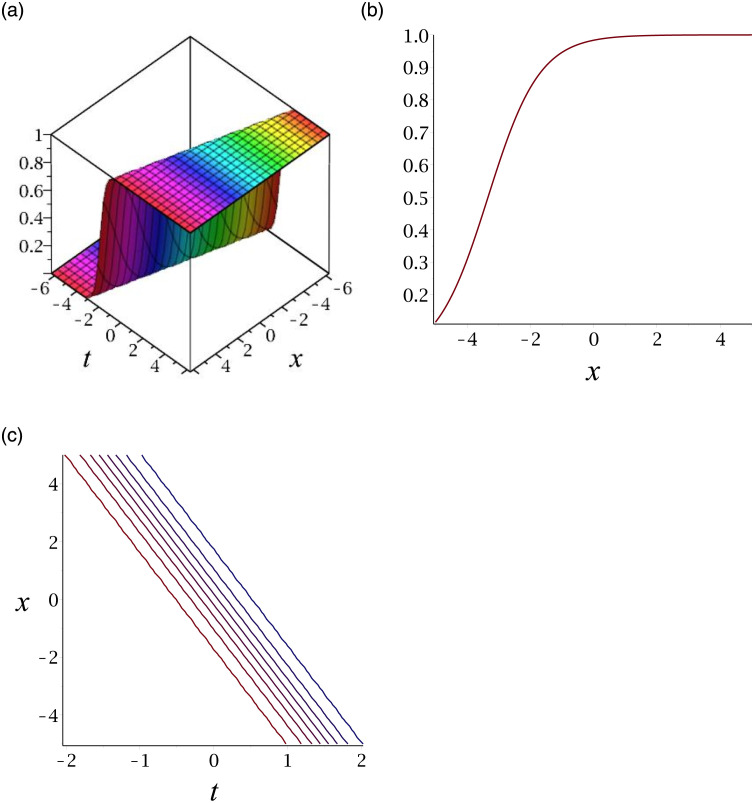
3D surface graphs, 2D plot and contour plot for |*v*_43_|.

Fig 14 depicts the anti-kink soliton for |*v*_43_|. Subplot (a) demonstrates the surface by taking *γ* = 3, *ϵ* = 1, *δ* = −0.5, *y* = 1, (b) represents the propagation of the wave in the direction of *x* − *axis*, whereas (c) shows its contour.

**Fig 15:**
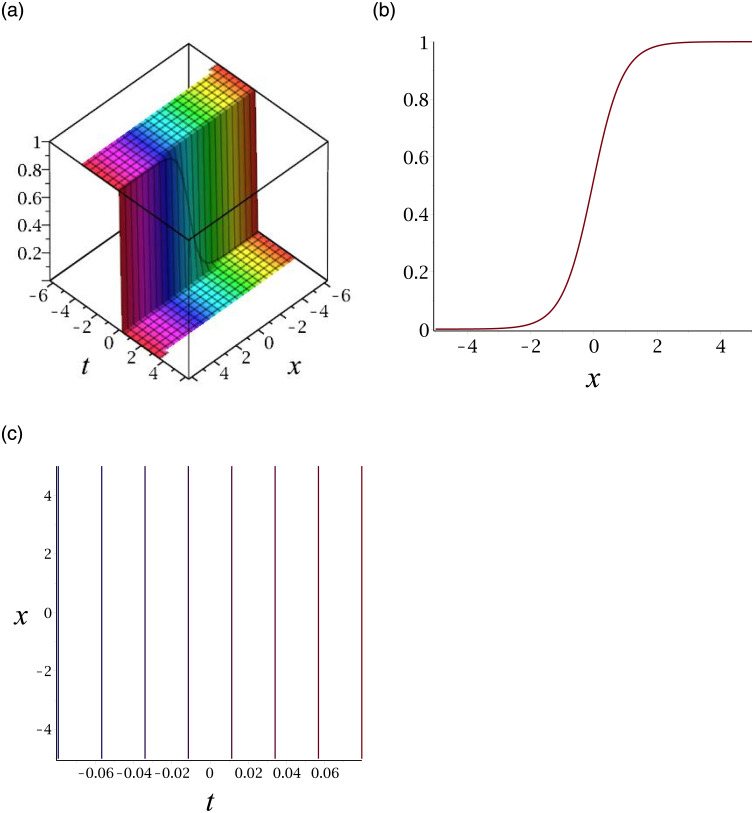
3D surface graphic and 2D plot and contour plot for |*v*_45_|.

Fig 15 depicts the kink soliton solution of |*v*_45_|. Subplot (a) demonstrates the surface for *γ* = 2, *ϵ* = 5, *δ* = −2, *y* = 1, (b) represent the propagation of the wave in the direction *x* − *axis*, whereas (c) shows its contour.

**Fig 16:**
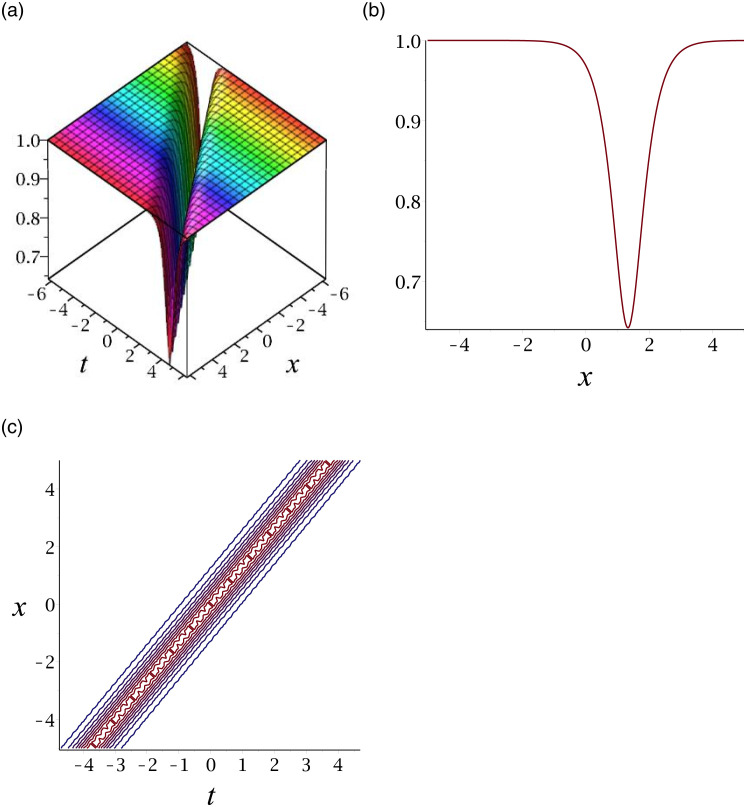
3D surface graphs, 2D plot and contour plot for *v*_47_.

Fig 16 depicts the solitery wave solution *v*_47_. Subplot (a) demonstrates the surface by taking *γ* = 3, *ϵ* = 2, *δ* = −2, *y* = 1, (b) represents the propagation of the wave in the direction of *x* − *axis*, whereas (c) shows its contour.

## 5 Conclusion

In the present study, different types of soliton solutions (bright, dark, singular, kink, anti-kink) and periodic wave solutions are obtained by using extended shGEET for (2+1)-dimensional Chaffee-Infante equation, which include trigonometric and hyperbolic functions. In addition, the 3D surface graphs, 2D line plots and contour plots are presented to understand the dynamics of the obtained solutions. It is concluded that extended shGEET is powerful, effective and convenient method to solve different nonlinear partial differential equations that arises in natural and applied sciences. The obtained results are hoped to be beneficial in the study of gas diffusion expressed by the (2+1)-dimensional CI equation.
